# Pilot Study: Impact of Primary Spoken Language as a Social Determinant of Health on CPR Education and Utilization

**DOI:** 10.5811/westjem.48504

**Published:** 2025-07-18

**Authors:** Charles W. LeNeave, Brian Meier, Heather Liffert, John C. Perkins

**Affiliations:** * Virginia Tech Carilion School of Medicine, Roanoke, Virginia; † Virginia Tech Carilion School of Medicine, Department of Emergency Medicine, Roanoke, Virginia; ‡ Carilion Clinic, Department of Emergency Medicine, Roanoke, Virginia; § Compress and Shock Foundation, Roanoke, Virginia

## Introduction

There are more than 350,000 out-of-hospital cardiac arrests every year in the United States. Neurologically intact survival is less than 10%. Recent literature shows that survival is even lower in communities of color and those that do not primarily speak English. Social determinants of health (SDOH), such as healthcare education access, language, and literacy, may serve as barriers to receiving cardiopulmonary resuscitation (CPR) education and using skills learned. Current literature is sparse on identifying which barriers may contribute to the lack of CPR education and utilization in non-English speaking communities. This study compared barriers to CPR education and utilization of CPR in English and Spanish-speaking learners. We hypothesized that language-specific barriers would be identified and may inform areas for further research. This study provides valuable insights into how CPR classes could be tailored to reduce disparities in CPR education and emergency access.

## Methods

In this cross-sectional, survey-based study, participants were recruited using convenience sampling at community-based events. These included free, non-certification, public CPR and automated external defibrillator (AED) classes, taught in English and/or Spanish, as well as non-medical gatherings in association with community organizations. Respondents were asked 10 closed-ended questions assessing the knowledge, comfort, and perceived barriers to CPR education, performing bystander CPR, and activating the 911 system. Survey responses were directly compared between language groups using fisher tests within R, adjusting for various sociodemographic factors.

## Results

A total of 307 surveys were collected, 179 in English and 128 in Spanish. Only 13% (n=16) of Spanish speakers stated they would have no concerns starting CPR, compared to 60% (n=107) in the English-speaking group. While the biggest barrier to initiating CPR in both groups was “fear of doing something wrong,” this was a much more common concern among Spanish speakers (50% vs. 26%). The language barrier was indicated by 33 (26%) Spanish speakers as a reason they would not give bystander CPR, compared to 0% in the English group. 79% (n=141) of English-speaking participants indicated they would have no problem calling 911, compared to only 16% (n=20) of Spanish-speaking subjects. Spanish speakers expressed substantially higher rates of concern over immigration status (8% vs 0.6%), fear of doing something wrong (16.5% vs 6.9%), and the language barrier (34.7% vs 1.7%), with regard to calling 911. Among participants with no prior CPR education, when asked why, Spanish speakers were more likely to believe they were unqualified (24% vs 10%) and cite cost as a critical factor (12% vs 2%).

## Conclusions

This study showed that Spanish speakers were less likely to know CPR or be comfortable initiating CPR than English speakers. While some barriers are common across language groups, Spanish speakers feel these more commonly. They are also burdened by barriers tied to SDOH, such as cost, language, and legal status. Having only 16% of a community being comfortable calling 911 is striking. These results suggest that marginalized communities will benefit from tailored educational models that address their unique challenges. Further research is necessary to better understand how SDOH serve as barriers to CPR education and CPR use.

